# Indian Hedgehog in Synovial Fluid Is a Novel Marker for Early Cartilage Lesions in Human Knee Joint

**DOI:** 10.3390/ijms15057250

**Published:** 2014-04-28

**Authors:** Congming Zhang, Xiaochun Wei, Chongwei Chen, Kun Cao, Yongping Li, Qiang Jiao, Juan Ding, Jingming Zhou, Braden C. Fleming, Qian Chen, Xianwen Shang, Lei Wei

**Affiliations:** 1 Department of Orthopaedics, the Second Hospital of Shanxi Medical University, Shanxi Key Lab of Bone and Soft Tissue Injury Repair, Taiyuan 030001, China; E-Mails: zang.cong.ming@163.com (C.Z.); weixiaochun11@126.com (X.W.); chenchongwei.ty@163.com (C.C.); caokun922@hotmail.com (K.C.); liyp328@126.com (Y.L.); jq1979@163.com (Q.J.); dd8052@163.com (J.D.); 2 Department of Orthopaedics, Warren Alpert Medical School of Brown University, CORO West, Suite 402A, 1 Hoppin Street, Providence, RI 02903, USA; E-Mails: jingmingzhou@gmail.com (J.Z.); braden_fleming@brown.edu (B.C.F.); qian_chen@brown.edu (Q.C.); 3 Department of Orthopaedics, Guiyang Medical University, No. 9 Beijing Road, Guiyang 550004, China; E-Mail: shangxianwen@medmail.com.cn

**Keywords:** Ihh, enzyme-linked immunosorbent assay, early cartilage lesions

## Abstract

To determine whether there is a correlation between the concentration of Indian hedgehog (Ihh) in synovial fluid (SF) and the severity of cartilage damage in the human knee joints, the knee cartilages from patients were classified using the Outer-bridge scoring system and graded using the Modified Mankin score. Expression of Ihh in cartilage and SF samples were analyzed with immunohistochemistry (IHC), western blot, and enzyme-linked immunosorbent assay (ELISA). Furthermore, we detected and compared Ihh protein levels in rat and mice cartilages between normal control and surgery-induced osteoarthritis (OA) group by IHC and fluorescence molecular tomography *in vivo* respectively. Ihh expression was increased 5.2-fold in OA cartilage, 3.1-fold in relative normal OA cartilage, and 1.71-fold in OA SF compared to normal control samples. The concentrations of Ihh in cartilage and SF samples was significantly increased in early-stage OA samples when compared to normal samples (*r* = 0.556; *p* < 0.001); however, there were no significant differences between normal samples and late-stage OA samples. Up-regulation of Ihh protein was also an early event in the surgery-induced OA models. Increased Ihh is associated with the severity of OA cartilage damage. Elevated Ihh content in human knee joint synovial fluid correlates with early cartilage lesions.

## 1. Introduction

Osteoarthritis (OA) is the leading cause of joint pain and disability in the elderly and characterized by progressive articular cartilage degeneration [1,2]. Since the progression of OA is a slow degenerative process, early diagnosis is critical for its prevention and treatment. The primary method to diagnose and assess OA progression is through radiography. Unfortunately, the relatively large errors and poor sensitivity of the method limit its use for early diagnosis and assessment of OA. There remains a need to identify patients in the early stages of disease before OA can be detected on radiographs so we may better understand the onset, progression and treatment of OA.

Identification of specific biological markers of articular cartilage metabolism to predict patients at risk for OA has received considerable attention [3,4]. Although many biomarkers from synovial fluid (SF), serum and urine have been explored, no reliable biomarkers for early diagnosis and identification of different OA stages have been validated for use in human [5]. Therefore, finding reliable biomarkers that can predict early OA and monitor the progression of the disease remains a challenge for investigators.

Normal articular cartilage chondrocytes maintain a “maturational arrested state” by molecular constraints. Nonetheless, chondrocytes can recapitulate some of the differentiation processes that occur in embryogenesis during OA development [6–10]. Some cartilage components that are only secreted by differentiating chondrocytes in the developmental growth plate begin to occur and increase in articular cartilage during the development of OA. For instance, type X collagen and MMP-13 are standard markers for chondrocyte hypertrophy during development, are not present in healthy articular cartilage, and their concentrations are increased in OA cartilage [11,12].

Indian hedgehog (Ihh) is one of three Hh ligands, specifically expressed by flattened prehypertrophic chondrocytes during embryonic development [13,14]. Ihh regulates chondrocyte hypertrophy and endochondral ossification [15,16]. Activation of Hh signaling pathways results in a decrease in articular cartilage thickness and proteoglycan (PG) content, while inhibiting Hh signaling leads to the opposite results in mice [17–19]. Several studies have demonstrated that the presence of Ihh in human and mice cartilage samples is positively associated with osteoarthritic cartilage damage [14,18]. Our previous study found that Ihh was induced in human OA cartilage and SF, and that the concentration of Ihh was associated with the extent of OA cartilage damage [14].

Our hypothesis is that monitoring changes in Ihh levels in the SF may provide reliable information on cartilage integrity. To test this hypothesis, we obtained SF samples from patients with OA and measured the concentration of Ihh by ELISA. We quantified cartilage damage using the Outer-bridge scoring system at time of surgery in these patients, and then evaluated the correlation between Ihh concentration in SF and cartilage damage. We observed that the levels of Ihh protein in SF were positively associated with scores that denoted early stage cartilage damage. We further compared the changes in Ihh protein levels in normal controls and surgically-induced-OA cartilage samples of rat and mice. Similarly to the human results, we found up-regulation of Ihh protein in early-stage OA cartilage in these animal models, suggesting this finding is conserved among human, rat, and mouse.

## 2. Results

### 2.1. Validation of Articular Cartilage Lesions in Human Knee Joints Using the Outer-Bridge Scoring System

**Figure 1.**
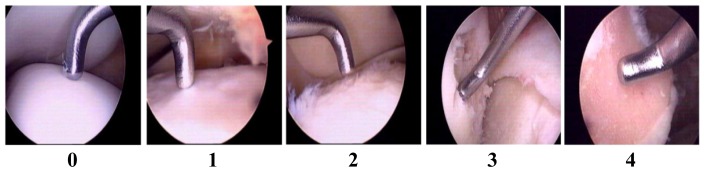
Articular cartilage lesion in human knee joint evaluated by arthroscopy with the Outer-bridge scoring system: (**0**) Normal cartilage; (**1**) Cartilage softened and/or swollen; (**2**) Cartilage fissures forming or already formed, but less than 50% of full thickness; (**3**) Fissures larger than 50%, but subchondrol bone not exposed; (**4**) Subchondral bone exposed or cartilage flakes detached.

In order to have uniform criteria for cartilage damage during arthroplasty and arthroscopy, the Outer-bridge scoring system was used to score the cartilage lesion of the knee joints (Figure 1).

### 2.2. Increased Indian Hedgehog (Ihh) Expression in Human Osteoarthritis (OA) Cartilage Determined by Immunohistochemistry (IHC) and Western Blot Analysis

**Figure 2.**
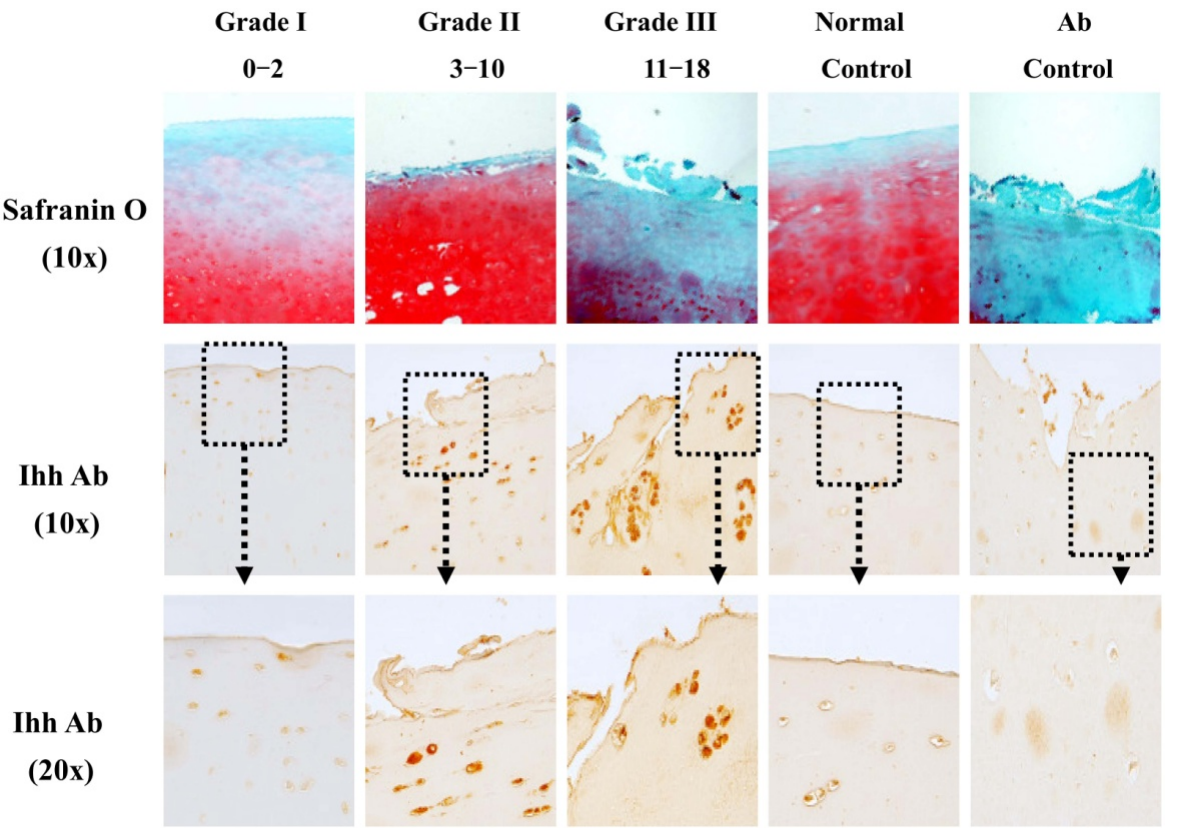
Increased Indian hedgehog (Ihh) expression in osteoarthritis (OA) cartilage determined by immunohistochemistry (IHC). IHC showed that the expression of Ihh is significantly increased in knee OA cartilage (*n* = 15, seven males, eight females, age 63.4 ± 7.1 (mean ± SD), range 54–78) compared to resection specimens with normal cartilage (*n* = 5, 4 males, 1 females, age 58.6 ± 7.7 (mean ± SD), range 49–69). A strong Ihh staining is seen in the upper layer of OA cartilage. Increased Ihh staining is associated with the severity of OA cartilage damage as demonstrated by Safranin O stain. In contrast, Ihh staining was minimal in normal cartilage. Grade I, Mankin score 0–2; Grade II, Mankin score 3–10; Grade III, Mankin score 11–18.

**Figure 3.**
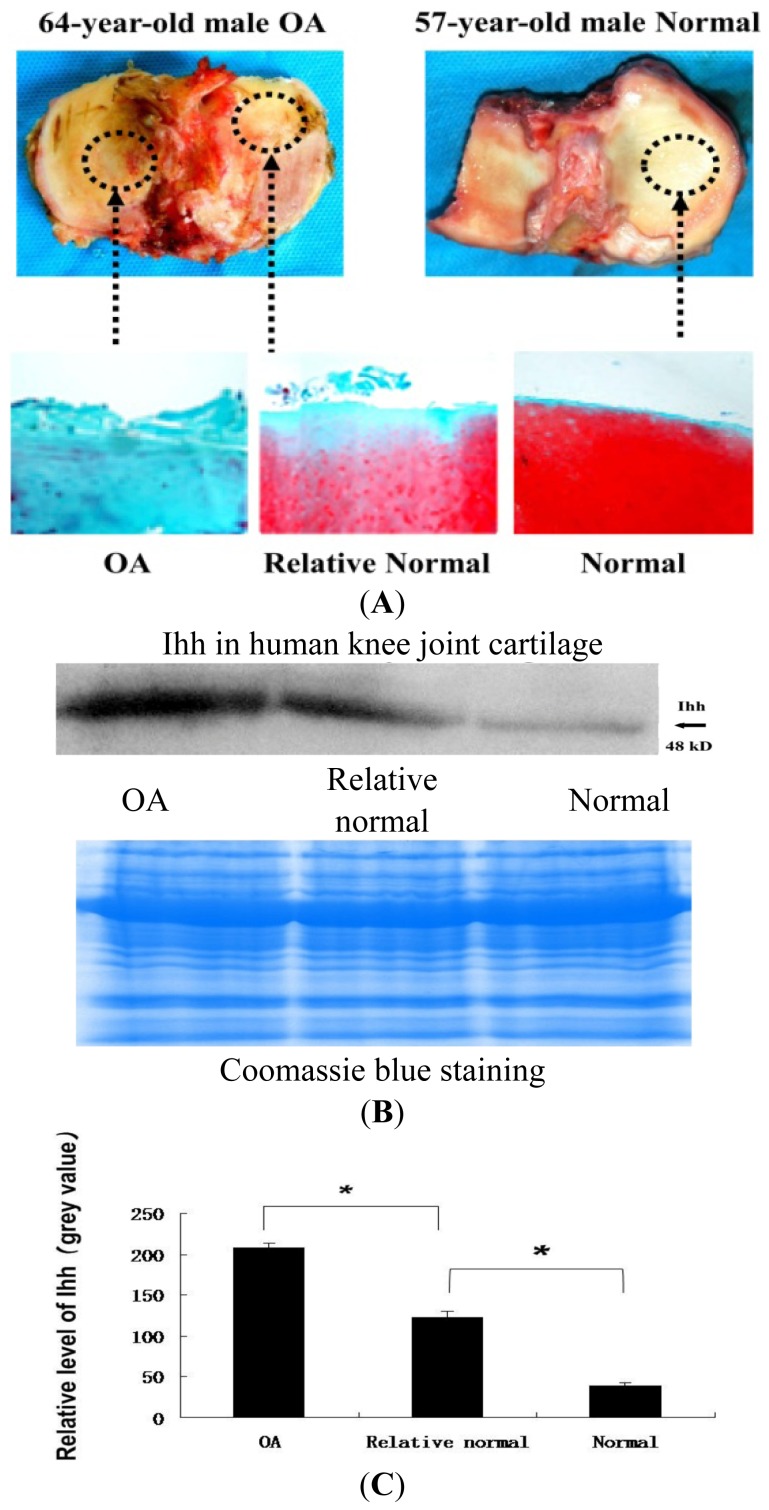
OA cartilage damage is associated with the increase of Ihh expression determined by western blot in cartilage. (**A**) Representative Safranin O stain from OA cartilage, adjacent relative normal and normal control cartilage, magnification 10×; (**B**) Western blot demonstrates the level of Ihh protein from OA, relative normal and normal cartilages, arguing that the increase of Ihh is associated with the severity of cartilage damage. Coomassie Blue stain was used to confirm equal loading; (**C**) Density of the Ihh band from Western blot was semi-quantified using the Image Analysis Software (Image Lab 3.0, Bio-Rad, Hercules, CA, USA). Bar graphs show the average with SD; *n* = 3; * *p* < 0.001; *p* value of less than 0.05 was considered statistically significant.

IHC (Figure 2) showed the expression of Ihh protein increased with the severity of articular cartilage damage and that the most intense staining for Ihh was mainly found in the upper clusters of cells in Grade III OA cartilage. When compared to OA cartilage, no obvious staining of Ihh was observed in the normal cartilage. Western blots (Figure 3) showed that the level of Ihh protein in OA cartilage was significantly higher than that in relative normal and normal control cartilages.

### 2.3. Increased Ihh Concentration in OA Synovial Fluid (SF) Determined by Western Blot

**Figure 4.**
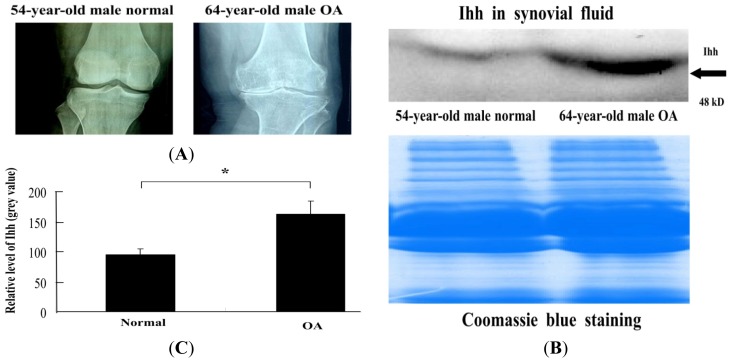
The increase of Ihh concentration found in OA SF. (**A**) Representative radiographs confirmed cartilage damage and joint space narrowing in the OA patients and no joint changes in the normal controls; (**B**) Representative Western blot demonstrates a high level of Ihh protein in human OA SF (64-year-old male) compared to normal control (54-year-old male healthy control). Coomassie Blue stain was used to confirm equal loading; (**C**) Gray value of Ihh band from Western blot was semi-quantified by Image Analysis Software (Image Lab 3.0). Bar graphs show the average with SD, *n* = 3, * *p* = 0.008.

SF samples were processed to determine Ihh content by western blot. Ihh concentration was 171% higher in SF samples collected from OA patients when compared to that in the normal controls (Figure 4).

### 2.4. Ihh Concentration in SF Was Dependent on Cartilage Damage as Determined by the Maximal Outer-Bridge Score

We found that the 122 patients could be divided into three sub-groups based on the modified Enshui [20] cartilage damage grading and synovial Ihh concentration (Table 1). The normal group included 25 patients with Outer-bridge grades of 0 who demonstrated normal cartilage.

**Figure 5.**
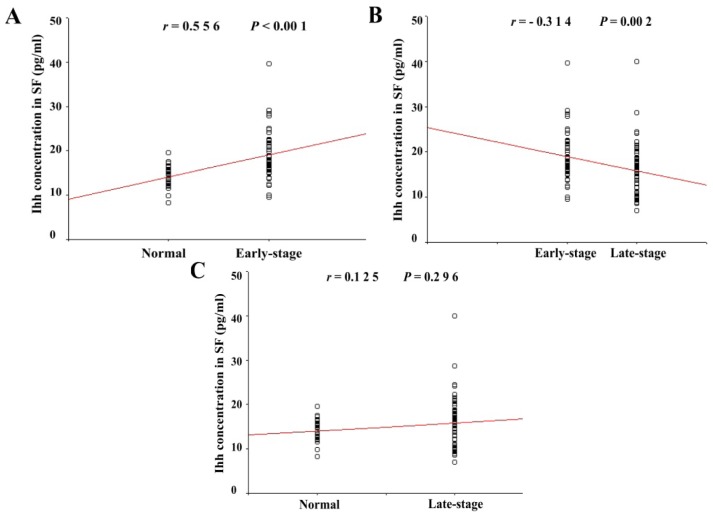
The increase of Ihh concentration in synovial fluid (SF) was correlated to the early stage of OA cartilage lesions. There was a significant correlation (*r* = 0.556; *p* < 0.001) between the Ihh concentration in SF and the articular cartilage Outer-bridge score from early stage OA (Outer-bridge 1–2, *n* = 50) but not in the late stage OA group (Outer-bridge 3–4, *n* = 47) compared to the normal control (Outer-bridge 0, *n* = 25). The level of Ihh was decreased in the late stage OA due to the severity cartilage damage. Spearman’s test was used for statistic analysis; *p* values of less than 0.05 were considered statistically significant.

The early-stage OA group included 50 patients with Outer-bridge grades of 1 and 2 who demonstrated slight cartilage erosion. The late-stage OA group included 47 patients with Outer-bridge grades 3 and 4 who demonstrated extensive cartilage erosion. The analysis showed that Ihh content in the SF of the patients from the early-stage OA group (19.03 ± 5.2 pg/mL) was significantly higher than that of the normal group (14.04 ± 2.55 pg/mL) (*p* < 0.001). A significant correlation was found between Ihh content and articular cartilage Outer-bridge score of the early-stage and normal groups (*r* = 0.556) (Figure 5). However, SF Ihh content in the late-stage group (15.16 ± 6.28 pg/mL) returned to the basal level and no statistically significant difference was detected between late-stage and normal groups (*p* = 0.296). The intra-assay and inter-assay variations of were lower than 8% and 10%, respectively.

### 2.5. The Elevated Ihh Signal Was also Detected in Surgery-Induced OA Models

**Figure 6.**
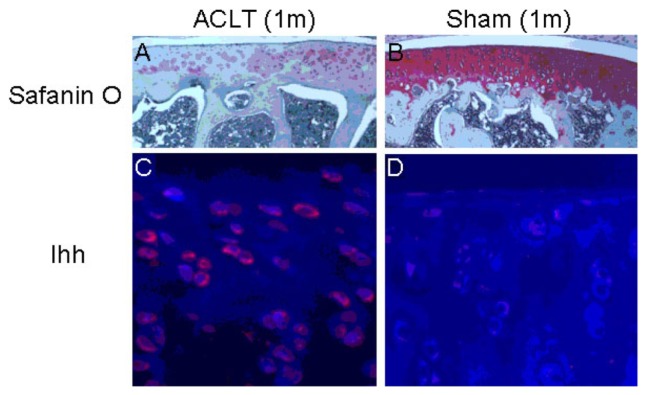
Elevated Ihh found in surgery-induced OA models. Elevated Ihh was found in a rat OA model induced by anterior cruciate ligament transection (ACLT) one month after surgery. In this model, there was decreased proteoglycan (PG) staining determined by Safranin O (**A**) compared with sham control (**B**) one month after surgery; immunohistochemistry (IHC) staining revealed increased Ihh in the surgically-induced OA cartilage (**C**) compared to sham (**D**). The expression of Ihh observed in the model is similar to that seen in human OA cartilage. 1m: 1 month after surgery.

Significant increases in Ihh expression were found in surgically-induced OA cartilage samples, along with dramatic decreases in cartilage PG staining (Figure 6), which was consistent with previous findings [21,22].

**Figure 7.**
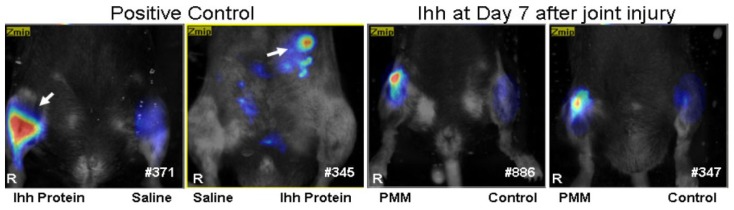
Elevated Ihh was also validated by Fluorescence Molecular Tomography (FMT) in our mouse partial medial meniscectomy (PMM) surgery-induced OA model. First, we validated whether Ihh-FMT probe can detect Ihh specifically *in vivo*. Two hundred ng Ihh recombinant protein was injected into right knee join and left belly. We found that a strong positive signal of Ihh was only detected at right knee and left belly (arrow) respectively but not in the saline injected sides (left knee and right belly) 24 h after injection (#371 and #345). A positive signal of Ihh was also detected at day 7 after right knee subjected to PMM joint injury but not in the left control knee (right two animals: #886 and #347, *n* = 5); R, right knee.

To further confirm the changes in Ihh after joint injury, Ihh antibody was conjugated to Vivo-Tag680™ NIR fluorochrome (PerkinElmer, Waltham, MA, USA) to create a probe for the detection of Ihh protein *in vivo*. We found the Ihh probe has a high specificity as the positive signal was only detected in the right knee joint and left belly where the 200 ng Ihh recombinant protein injection was performed 24 h previously (Figure 7). No positive signal was detected in the left knee joint and right belly where equal amounts of saline were injected as control. The positive signal detected by Ihh probe was found 7 days after partial medial meniscectomy (PMM) surgery in the right knee but not in the left control knee (Figure 7, right two mice, *n* = 5).

## 3. Discussion

Currently, assessment of cartilage lesions mainly depends on the detection of joint space narrowing by radiography. This method cannot detect early cartilage damage due to poor sensitivity and a relatively large precision error, nor has it been used to associate with symptoms or functional disability [23–25]. Thus, the development of assays for biochemical biomarkers using metabolites and degradation products of cartilage has potential therapeutic value as a tool to detect early cartilage damage and allow early intervention.

Previous reports found that at least a portion of the chondrocytes in OA cartilage, exit a permanent stage and enter differentiation cycle and show hypertrophy-like changes, which resemble those of developmental growth plate chondrocytes [26,27]. Chondrocyte differentiation was closely related to the very early development of cartilage degeneration occurring in OA [28]. In the developing cartilage, Ihh is primarily expressed by prehypertrophic chondrocytes as an initial step during chondrocyte differentiation and hypertrophy. Therefore, Ihh expression may be a very early event in the cartilage degradation process of OA. Recent findings in human cartilage support this idea [17]. Further, recent studies report Hh signaling as involved in mouse OA development [18,29], and increased expression of type X collagen, a hypertrophic marker, in human knee joint cartilage with early focal OA-like lesions [10,28]. As Ihh is undetectable in healthy articular cartilage [30], monitoring changes in Ihh may provide information on the progression of OA. Our study illustrated that Ihh expression was positively associated with the severity of cartilage damage (Figure 2), consistent with previous findings [14]. This association suggests that Ihh expression in human cartilage may be a candidate biomarker for diagnosis of early OA and monitoring of OA progression. Studies are now needed to validate its effectiveness longitudinally and to demonstrate cause and effect.

Ihh is not only expressed by chondrocytes, but also by other cells such as osteoblasts [31]. Therefore, the Ihh content of serum and urine may not specifically reflect changes in cartilage metabolism. Metabolites or degradation products secreted by chondrocytes are directly released into the SF. As opposed to those in serum and urine, the biomarkers in SF of joints undergoing cartilage degeneration directly correlate with pathological changes in cartilage. Detection of the Ihh concentration in SF appears to be a promising method to diagnose early OA and monitor progression of OA cartilage damage in clinical patients.

In this study, we detected a significant elevation in Ihh level in the SF samples from OA knee joints compared to the control samples by western blot. This result is in agreement with a previous report [14]. We then used ELISA to quantity synovial Ihh concentration, and thusly established a positive correlation between synovial Ihh content and cartilage damage as graded with the Outer-bridge grading system that is considered the “gold standard” for assessment of articular cartilage lesions and is repeatable between surgeons [23].

The content of Ihh in the knee SF is low when the cartilage surface is intact. However, synovial Ihh content is dramatically increased once damage of cartilage surface occurs. This finding suggests that the synovial concentration of Ihh could potentially serve as a diagnostic biomarker for early damage of articular cartilage. This observation also indicates that cartilage superficial zone may act as the “protective screen” for cartilage integrity, and that once superficial zone has fissures, inflammatory factors and harmful cytokines may have a direct stimulatory effect on chondrocyte differentiation implicated in pathological development of OA cartilage. Consequently, Ihh expression is rapidly upregulated with an increase of the hypertrophic chondrocytes marker, type X collagen.

Interestingly, our study also found that synovial Ihh concentration in late-stage cartilage returned to basal levels comparable to those in normal cartilage. Many previous studies have independently demonstrated that there is a significant decrease in chondrocyte numbers in articular cartilage during aging [32–36], while some other reports found positive correlations between severity of cartilage damage and chondrocyte death due to apoptosis [37]. Thus, one possible explanation is that synovial Ihh decreases in late-stage OA due to the reduction in chondrocyte numbers and deficient in cartilage.

In this study, we also found that the level of Ihh protein was up-regulated in rat cartilage with ACL transected-induced OA. Increased Ihh expression was also observed in early-stage OA cartilage degeneration induced by the PMM in a mouse model, similar to what happens in humans. These observations suggest that the Ihh signaling pathway in the joint cartilage is conserved between human and animal models.

In conclusion, the level of the Ihh in the human knee SF could potentially be used as an early biomarker to reflect cartilage damage, however it may not be sensitive enough to predict the severity and progression of knee articular cartilage damage in the later stages of disease, a point in time at which radiographic imaging is sufficient.

## 4. Experimental Section

### 4.1. Enrollment of Patients

This study was approved by the Institutional Review Board at the second Hospital of Shanxi Medical University (Taiyuan, China; CMTT#: 2013025. Approval 3 June 2013) and informed consent was obtained from each donor. The study enrolled 127 patients (62 males and 65 females) with an average age of 40.2 years (40.5 ± 15). Among these patients, 40 received total knee replacement because of OA, and 87 underwent arthroscopy because of meniscus injury (*n* = 30), anterior or posterior cruciate ligament injury (*n* = 30), arthroscopic debridment due to pain and disability from OA (*n* = 21), loose body removal (*n* = 1), and amputation due to severe trauma (*n* = 5). OA diagnosis was made by clinician’s assessment using American College of Rheumatology (ACR) criteria [38]. Patients who had inflammatory joint disease, acute major trauma, malignant tumors or abnormal renal and liver function were excluded. Patients who were treated with corticosteroids within the 3 months preceding surgery were also excluded.

### 4.2. Cartilage Tissue Samples

Cartilage samples from the tibia obtained during total knee arthroplasty were divided into two categories: (1) OA cartilage from the more affected compartment (usually medial); and (2) “relatively normal” cartilage from the uninvolved compartment (usually lateral). Age-matched normal cartilage samples were also obtained from knee joints immediately following amputation due to severe trauma. Absence of cartilage degeneration was confirmed in the normal cartilage samples using Safranin O staining.

### 4.3. Synovial Fluid Analysis

A volume of 0.5–5 mL of SF was aspirated from the knee joint just before total knee replacement or arthroscopy. The SF sample was immediately centrifuged for 15 min at 3000 rpm to remove particulate material, and the supernatant was aliquoted, rapidly frozen, and stored at −70° C until analysis. Before freezing, the SF samples were diluted 1:5 with cell lysis buffer containing proteinase inhibitor (Roche, Basel, Switzerland).

### 4.4. Animals

Sections of OA cartilage from a rat OA model induced by anterior cruciate ligament transection (ACLT) were a subset of those used previously [22]. In this model, animals showed early OA changes one month after surgery [21,22]. The mouse model consisted of 2-month-old C57 BL/6 male mice that underwent partial medial meniscectomy (PMM) surgery. These mice underwent Fluorescence Molecular Tomography (FMT) 7 days after PMM to study Ihh changes *in vivo*. All experiments were approved by the Animal Care and Use Committee of Rhode Island Hospital. (CMTT#: 0111-12. Approval 6 December 2012).

### 4.5. Histology

Full thickness 1 × 1 cm cartilage samples were taken from the OA cartilage and adjacent “relatively normal” cartilage samples from each knee joint with a scalpel. The sections (0.2–0.3 g) were fixed in 10% formalin (Sigma-Aldrich, St. Louis, MO, USA) for 72 h, decalcified in RichmaneGelfand-Hill solution, processed in a Tissue-Tek VIP 1000 tissue processor (Miles, Elkhart, IN, USA), and embedded in a single block of Paraplast X-tra (Thermo-Fisher, Hampton, NH, USA). Blocks were trimmed to expose tissue using a rotary microtome (Leica, Wetzlar, Germany). The slices were then cut into 6-μm sections and mounted on slides. Safranin O stain was performed and the severity of cartilage damage was assessed using the modified Mankin grading system [17]. Grade I (Mankin score 0–2) represents minimal cartilage damage, while Grades II (Mankin score 3–10) and III (Mankin score 11–18) represent more severe cartilage damage. Three independent and blinded observers scored each section, and the scores were averaged. Early OA changes from the previous rat ACLT study [21,22] were validated using Safranin O stain.

### 4.6. Immunohistochemistry (IHC)

To detect the distribution of Ihh in human cartilage, 6-μm sections were collected and mounted on positively charged glass slides (Thermo-Fisher). The sections were dried on a hot plate to increase adherence to the slides. IHC was carried out using the Histostain-SP Kits (Zymed-Invitrogen, Carlsbad, CA, USA). Slides were deparaffinized and rehydrated through conventional methods and blocked in 3% hydrogen peroxide (Sigma-Aldrich, St. Louis, MO, USA) in methanol (Sigma-Aldrich, St. Louis, MO, USA) for 30 min. Nonspecific binding was blocked in 15% normal serum (LICOR, Lincoln, NE, USA) matched to the secondary antibody species. The sections were digested by 5 mg/mL hyaluronidase (HA) in PBS (Sigma-Aldrich) for 20 min. Slides were incubated overnight at 4 °C with with a polyclonal antibody against Ihh at 1:500 dilution (sc-1196, Santa Cruz, Santa Cruz, CA, USA). The negative control sections were incubated with isotype control (sc-1196-P, Santa Cruz) in PBS. The specificity of Ihh antibodies used in this study has been validated by immunofluorescence stain and Western blot [14]. Thereafter, the sections were treated sequentially with biotinylated secondary antibody and streptavidin-peroxidase conjugate (Zymed-Invitrogen), and then were developed in DAB chromogen (Zymed-Invitrogen). The sections were counterstained with hematoxylin (Zymed-Invitrogen). Photography was performed with a microscope (BX51, Olympus, Tokyo, Japan). In the rat ACLT model, the cartilage degeneration occurs within one month after surgery [21,22]. To test whether there is a change of Ihh following ACL injury, the expression of Ihh was determined by IHC one month after ACLT in this model as published before [14].

### 4.7. Western Blot

Total protein in cartilage and SF samples was quantified using the BAC Protein Assay Reagent Kit (Pierce, Rockford, IL, USA). Fifty micrograms of total protein was electrophoresed in 10% SDS-PAGE under reducing conditions. After electrophoresis, proteins were transferred onto Immobilon-Polyvinylidene Difluoride (PVDF) membrane (Beyotime, Shanghai, China) and probed with a polyclonal antibody against Ihh (Santa Cruz). The antibody was diluted 1:500 in TBS-T containing 1% bovine serum albumin (BSA) (Sigma Aldrich). Horseradish peroxidase-conjugated second antibody IgG (Santa Cruz) was diluted 1:1000 in TBS-T. Visualization of immunoreactive proteins was achieved by using the ECL Western blot detection reagents (CWBIO Corporation, Beijing, China) and by subsequently exposing the membrane to Molecular Imager (Bio-Rad, Hercules, CA, USA). Band densities were quantified using Image Acquisition and Analysis Software (UVP, Upland, CA, USA). Parallel gels were prepared for Coomassie Blue stain to confirm equal loading of samples. Equal amounts of protein were electrophoresed in 10% SDS-PAGE and the gel was prefixed in 50% methanol, 10% acetic acid, 40% dH_2_O for 30 min and then stained with 0.25% Coomassie Brilliant Blue R-250 (Beyotime, Shanghai, China) in the above solution for 15 min. The gel was detained in 25% methanol, 8% acetic acid, 67% dH_2_O until background was clear. The detained gel was stored in 7% acetic acid and a photograph was taken using Molecular Imager (Bio-Rad).

### 4.8. Evaluation of Cartilage Damage

In order to have uniform criteria for cartilage damage during arthroplasty and arthroscopy, we used the Outer-bridge scoring system. Articular cartilage lesions were evaluated during arthroscopy or via direct surgical observation using the Outer-bridge scoring system [39]. The absence of morphological changes on the articular cartilage scores 0 in the Outer-bridge scoring system, and softened or swollen articular cartilage scores 1. Once fissures smaller than 1.3 cm appear on the articular cartilage surface, a score of 2 is assigned, while fissures larger than 1.3 cm merit a score of 3. When cartilage flakes are detached from the articular surface and the subchondral bones are exposed, the Outer-bridge score is 4 [39]. Each of the six regions of the knee cartilage (*i.e.*, patellar, femoral groove, medial femoral condyle, lateral femoral condyle, medial tibial plateau, and lateral tibial plateau were assigned the appropriate Outer-bridge score (Figure 1). The highest score recorded within a knee joint was defined as the final grading.

### 4.9. Measurement of Synovial Ihh

The concentration of Ihh in SF was measured with a competitive polyclonal antibody-based ELISA kit (CUSABIO Corporation, Wuhan, China). After thawing to room temperature, samples were diluted at a 1:4 ratio, and 100 μL of the diluted sample together with 100 μL of biotinylated anti-Ihh antibody were added to each well of a 96-well plate. After 2 h incubation at 37 °C, the wells were drained and washed 3 times with washing buffer, and 100 μL of streptavidin-HRP solution was then added for 1 h incubation at 37 °C. Substrate A and B solutions were added to each well, followed by incubation for 10 min at 37 °C before the reaction was stopped by 50 μL of sulfuric acid. The absorbance was read by a spectrophotometer (Packard FluoroCount BF10000, eBay, San Jose, CA, USA) at 450 nm wavelength. All samples were measured in triplicate, and the average reading was recorded.

### 4.10. Fluorescence Molecular Tomography (FMT)

FMT enables real-time 3D detection of fluorochrome distribution in tissues of live animals [40–43]. To further confirm the changes of Ihh after joint injury, Ihh antibody was conjugated to Vivo-Tag680™ NIR fluorochrome to create a probe for the detection of Ihh protein *in vivo* in the PMM mouse OA model 7 days after surgery. Mice were anesthetized with an intraperitoneal injection of ketamine (75 mg/kg) and medetomidine (1 mg/kg), placed in an upright position in the imaging chamber, and then imaged with the FMT system (ViSen, Waltham, MA, USA). A Near-infrared (NIR) laser diode emitting continuous wave radiation at wavelengths of 670 nm trans-illuminated the lower body of each animal from posterior to anterior, and both excitation and emission signals were detected by a charge-coupled device (CCD) camera and appropriate band pass filters. Ihh antibody probes in the knee joint were determined using Region of Interest (ROI) analysis.

### 4.11. Statistical Analysis

Differences in Ihh band densities from OA, relatively normal and normal cartilage samples were compared using one-way ANOVA. A *t*-test was also used to compare Ihh band densities from OA SF to normal SF at a rejection level of 5% unless otherwise noted. Nonparametric Spearman rank correlation coefficient was used to examine the relationships between cartilage erosion and Ihh concentration in SF. All data from ELISA are expressed as mean ± SD, and analyzed using SPSS 13.0 software (SPSS Inc., Chicago, IL, USA). One-way ANOVA was used for among group comparisons. *p* values less than 0.05 were considered statistically significant.

## 5. Conclusions

The level of Ihh in SF from early cartilage damage groups was significantly higher than in the control groups, but no significant difference was detected in the level of Ihh among the severely damaged cartilage sub-groups. There was a significant correlation between the Ihh concentration in the synovial fluid and outer-bridge scores (*r* = 0.556) from early stage cartilage damage pateints. Thus, elevated Ihh level is correlated with early cartilage lesions, but it may not be sensitive enough to predict the progression of severity of cartilage damage in the knee joint.

## Figures and Tables

**Table 1.** Indian hedgehog (Ihh) concentration in SF. Results are presented as the mean ± SD.

Group (Based on Outer-Bridge Score)	Ihh Concentration (pg/mL)	*n*
Normal Group	14.04 ± 2.55	25
Early stage Group	19.03 ± 5.2	50
Late stage Group	15.16 ± 6.28	47

Total		122

*n*, number; Normal group, articular cartilage Outer-bridge score = 0; Early stage group, articular cartilage Outer-bridge score = 1–2; Late stage group, articular cartilage Outer-bridge score = 3–4.
